# Comparative evaluation of thermography, infrared, mercury, digital, and ecological thermometers for body temperature measurements in cattle

**DOI:** 10.1007/s11250-026-05059-w

**Published:** 2026-05-12

**Authors:** Ana Carolina Pinheiro, Rodrigo Siuffi Abbud, Kelly Grayce Perestrelo, Jennifer Evangelista de Amorim, Julia Marques Nascimento Freitas, Maria Claudia Araripe Sucupira

**Affiliations:** Department of Clinical Medicine, School of Veterinary Medicine and Animal Science, University of São Paulo, São Paulo, Brazil

**Keywords:** Infrared thermography, Digital thermometer, Galinstan thermometer, Cattle, Body temperature, Mercury replacement

## Abstract

Following the global ban on mercury thermometers due to their toxicological risks, alternative devices such as digital, ecological (galinstan-based), and infrared thermometers, as well as infrared thermography, have emerged for veterinary use. This study aimed to evaluate the accuracy and agreement of these alternatives compared to the mercury thermometer for measuring body temperature in cattle. Twenty-four clinically healthy or stable cattle were monitored twice daily over five consecutive days. Rectal temperatures were recorded using mercury, digital, and ecological thermometers. Infrared thermometers and thermographic cameras were used to assess cutaneous temperatures at the forehead, eyes, axillae, and perineum. Correlation analyses (Pearson or Spearman) and Bland–Altman plots were applied to determine agreement. Infrared thermography and infrared thermometer measurements at the eyes and perineum exhibited the highest correlations but failed to meet clinical agreement standards. In contrast, both ecological and digital thermometers showed strong correlation (*r* ≥ 0.85) and acceptable agreement limits with the mercury thermometer (mean difference < 0.3 °C; SD < 0.5 °C), indicating their suitability as substitutes in clinical practice. Despite the promise of infrared techniques for non-invasive screening, further validation is needed before clinical implementation.

## Introduction

Fever is a fundamental physiological response to infection or inflammation, mediated by pro-inflammatory cytokines acting on thermoregulatory centers in the hypothalamus (Li et al. 2020). Accordingly, body temperature measurement is a key diagnostic parameter in livestock health monitoring, and even modest increases in rectal temperature (≈ 1 °C) may result in productivity losses, particularly in heat-sensitive species such as cattle (McDowell et al. 1976; Salles et al. 2016).

Mercury-in-glass thermometers have long been considered the gold standard for core body temperature measurement due to their accuracy and reliability (Burfeind et al. 2010). However, concerns regarding mercury toxicity and environmental contamination have led to restrictions or bans in several countries, including those of the European Union, promoting the widespread adoption of digital thermometers in both human and veterinary medicine (Kreissl and Neiger 2015; Pecoraro et al. 2021).

Non-invasive infrared technologies, including handheld infrared thermometers and thermographic cameras, have gained attention as rapid, contactless tools for estimating body surface temperature (Hoffman et al. 2023; Katsoulos et al. 2016). However, surface temperature measurements are highly influenced by environmental conditions such as airflow, ambient temperature, and humidity, which may compromise their consistency (Church et al. 2014).

Ecological thermometers based on galinstan—a non-toxic alloy of gallium, indium, and tin—have also emerged as mercury-free alternatives that preserve the mechanical measurement principle of mercury thermometers (Baura 2021). Although their clinical equivalence to mercury devices has been demonstrated in human medicine (Dante et al. 2021), evidence supporting their application in veterinary practice remains limited.

Therefore, this study aimed to evaluate and compare the performance of digital, ecological, infrared, and thermographic thermometers for estimating body temperature in cattle, using mercury thermometers as the reference standard.

## Materials and methods

### Animals and experimental design

The study was conducted using 24 cattle of varying breeds, ages, and sexes from veterinary facilities and research institutions in Brazil. Animals were clinically healthy or in stable condition based on physical examination, and none received any medication during the study period.

Age distribution was as follows: 45% were ≤ 1 year old, 33% were between 1 and 3 years, and 22% were over 3 years. Body temperature was measured twice daily (7:00–9:00 a.m. and 1:00–3:00 p.m.) for five consecutive days, totaling 240 observations per thermometer.

### Environmental conditions

Environmental temperature and relative humidity were recorded at each session using a digital thermometer–hygrometer (HTC-1^®^, Underbody) and used for infrared thermography calibration (Church et al. 2014). Measurements were conducted in closed hospital rooms, covered calf pens, or shaded handling areas, all protected from direct solar radiation and without mechanical ventilation. Animals were positioned in sheltered areas to minimize airflow and convective heat loss.

Animals were handled calmly, were not exercised or exposed to direct sunlight prior to evaluation, and remained at rest for at least 10 min before thermographic measurements. Immediately prior to temperature acquisition, each animal was individually restrained, and all thermometric measurements were performed sequentially without prolonged containment. When necessary, calves were gently restrained using ropes, ensuring minimal handling. Although physical surroundings varied, key factors affecting infrared thermography—airflow, solar radiation, physical activity, and animal agitation—were consistently minimized across all sites.

### Measurement procedures

Each animal underwent a standardized sequence of measurements: (1) infrared thermography (IRA) using a FLIR T620 (FLIR Systems, Wilsonville, OR, USA); (2) infrared thermometer (INFRA) using a RESTAR^®^ infrared thermometer (SENSTECH LTD., Shenzhen, China); and (3) rectal thermometry using a standard mercury clinical thermometer (manufacturer not specified), a G-TECH^®^ TH1027 digital thermometer (Joytech Healthcare Co., Ltd., Hangzhou, China), and an Incoterm^®^ Clinical Ecological Thermometer 1.0 (Incoterm, Porto Alegre, Brazil). Infrared measurements were conducted before rectal thermometry to prevent handling-related interference, and the order of rectal thermometers was randomized.

**Fig. 1:**
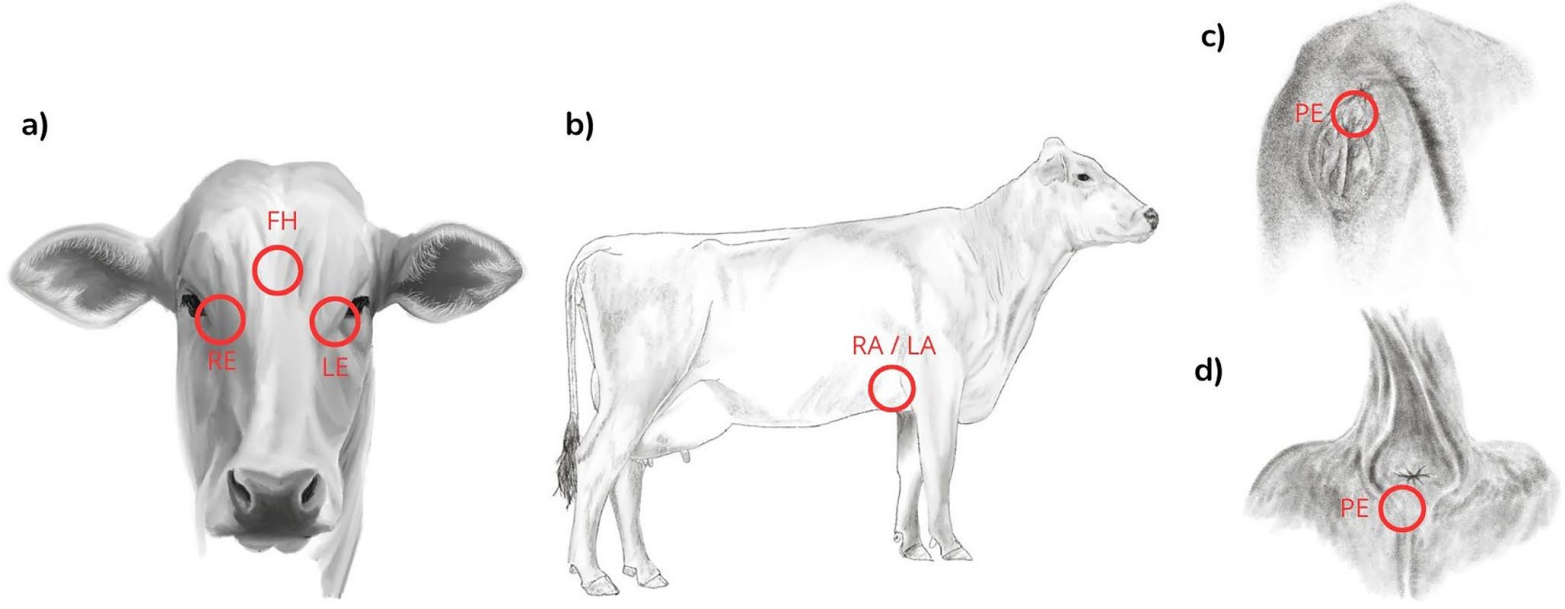
Anatomical locations used for infrared measurement. (**a**) Forehead (FE), lacrimal gland (RE = right eye; LE = left eye); (**b**) Axillae (RA = right axilla; LA = left axilla); (**c**) Perineum (PE) in females; (**d**) perineum (PE) in males

**Fig. 2:**
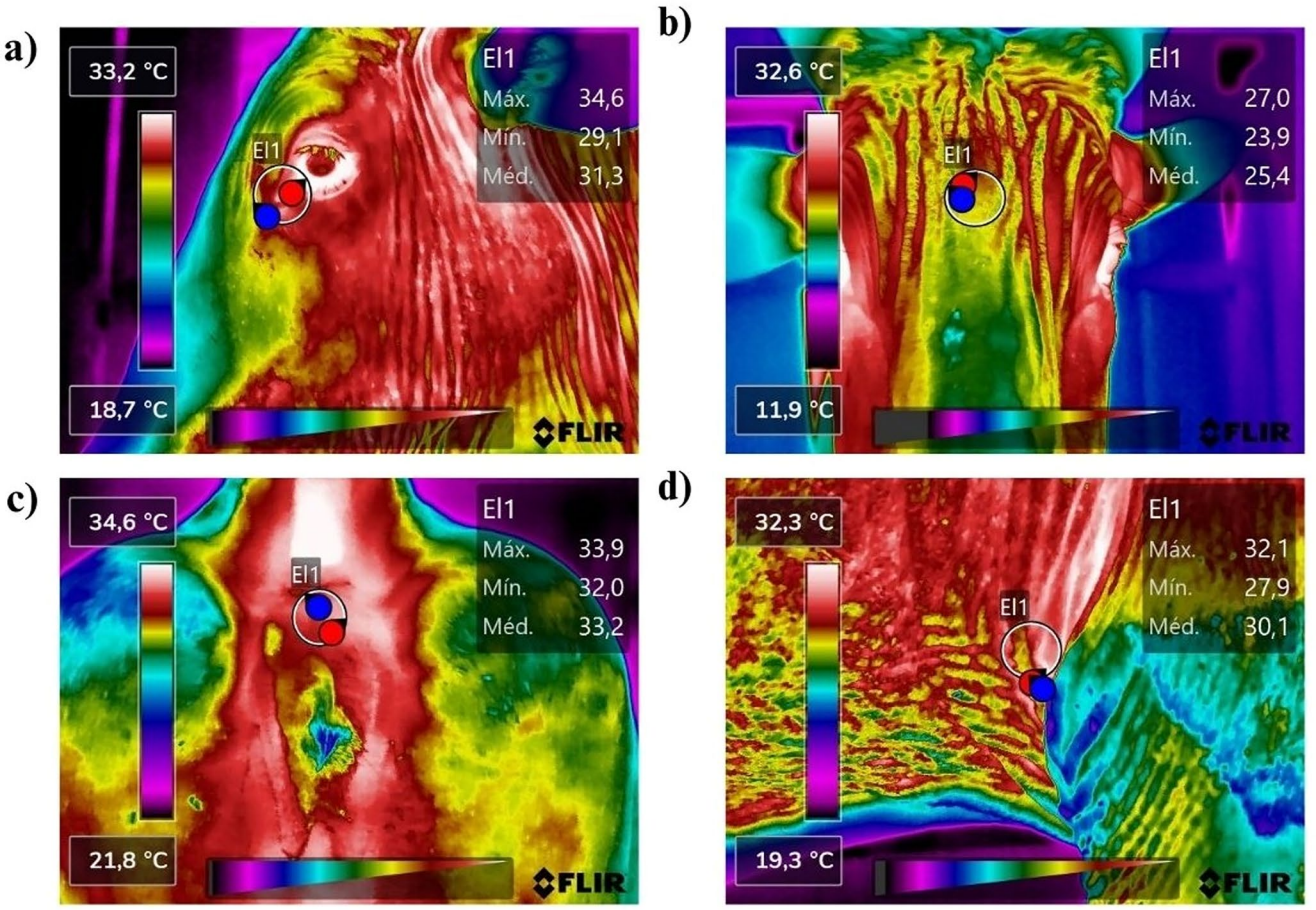
Infrared thermography images captured from FLIR Thermal Studio Software. (**a**) Left lacrimal gland; (**b**) Forehead; (**c**) Right axilla; (**d**) Perineum

Infrared measurements were obtained from six anatomical sites (Fig. 1): forehead (FH), right and left lacrimal glands (RE, LE), right and left axillae (RA, LA), and perineum (PE). Thermographic images were captured at 1 m from the animal, at a 45–90° angle, with emissivity set to 0.98 and environmental data entered into FLIR Thermal Studio software (Fig. 2). A circular region of interest (1.5 cm diameter) was applied to each site to extract maximum surface temperature for analysis (Johnson et al. 2011; Peng et al. 2019).

**Fig. 3:**
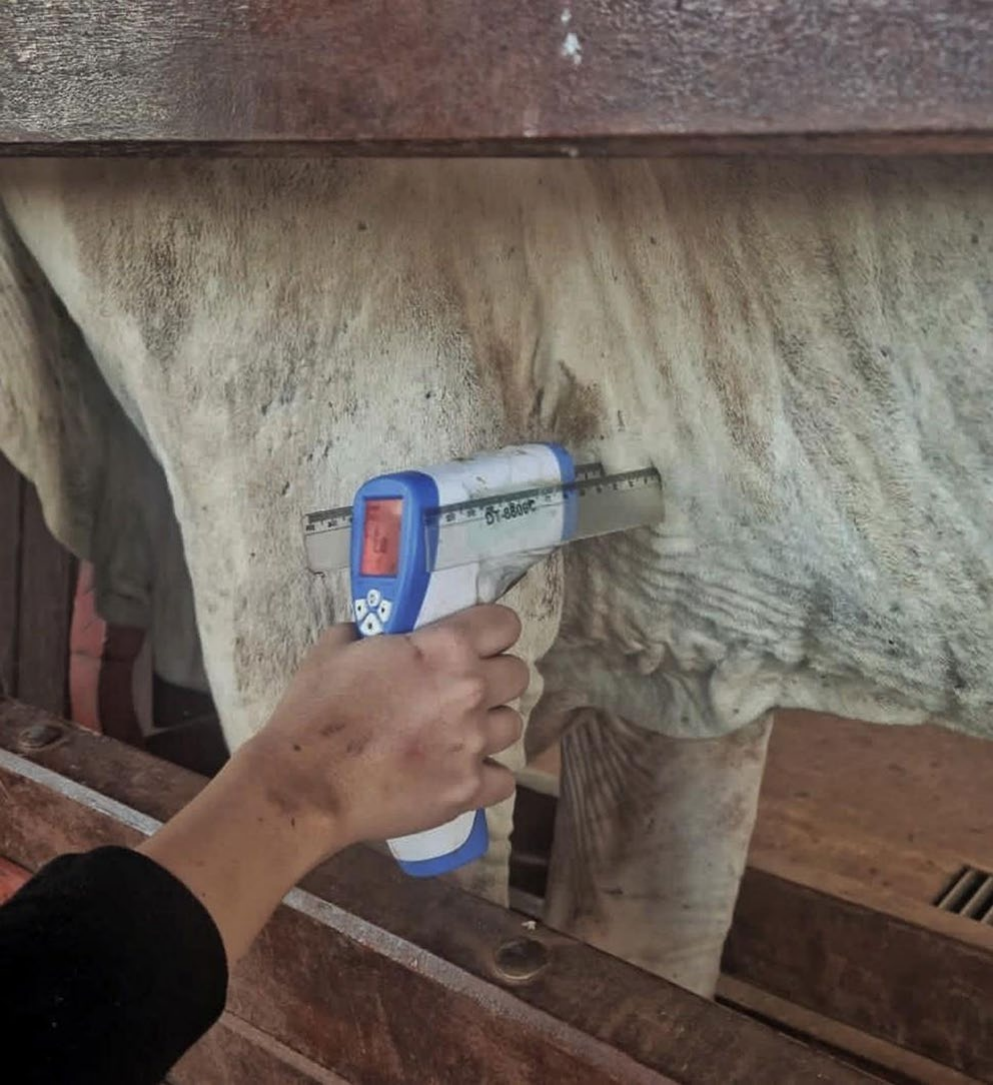
Infrared thermometer measurement performed in the left axilla region at a standardized distance of 5 cm using a ruler. The device display shows a “low” reading (< 33.8 °C), illustrating a frequent limitation observed during infrared thermometry under field conditions

**Fig. 4:**
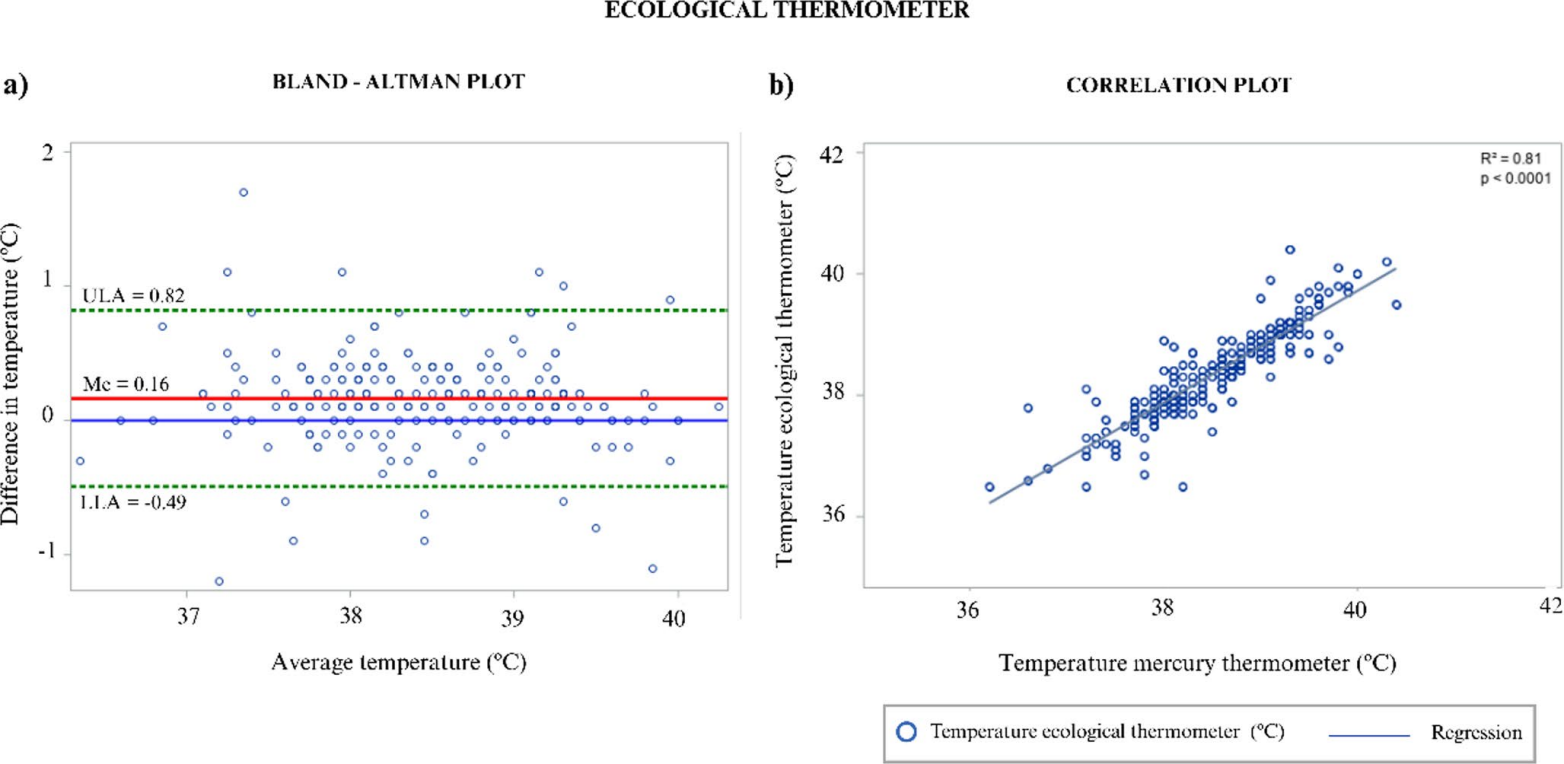
Ecological thermometer graphics. (**a**) Bland-Altman plot showing agreement between ecological and mercury thermometers (∆ = MER – ECO) in 24 cattle (*n* = 238). (**b**) Correlation plot with fitted linear regression line between ECO and MER (R² = 0.81, *p* < 0.0001)

**Fig. 5:**
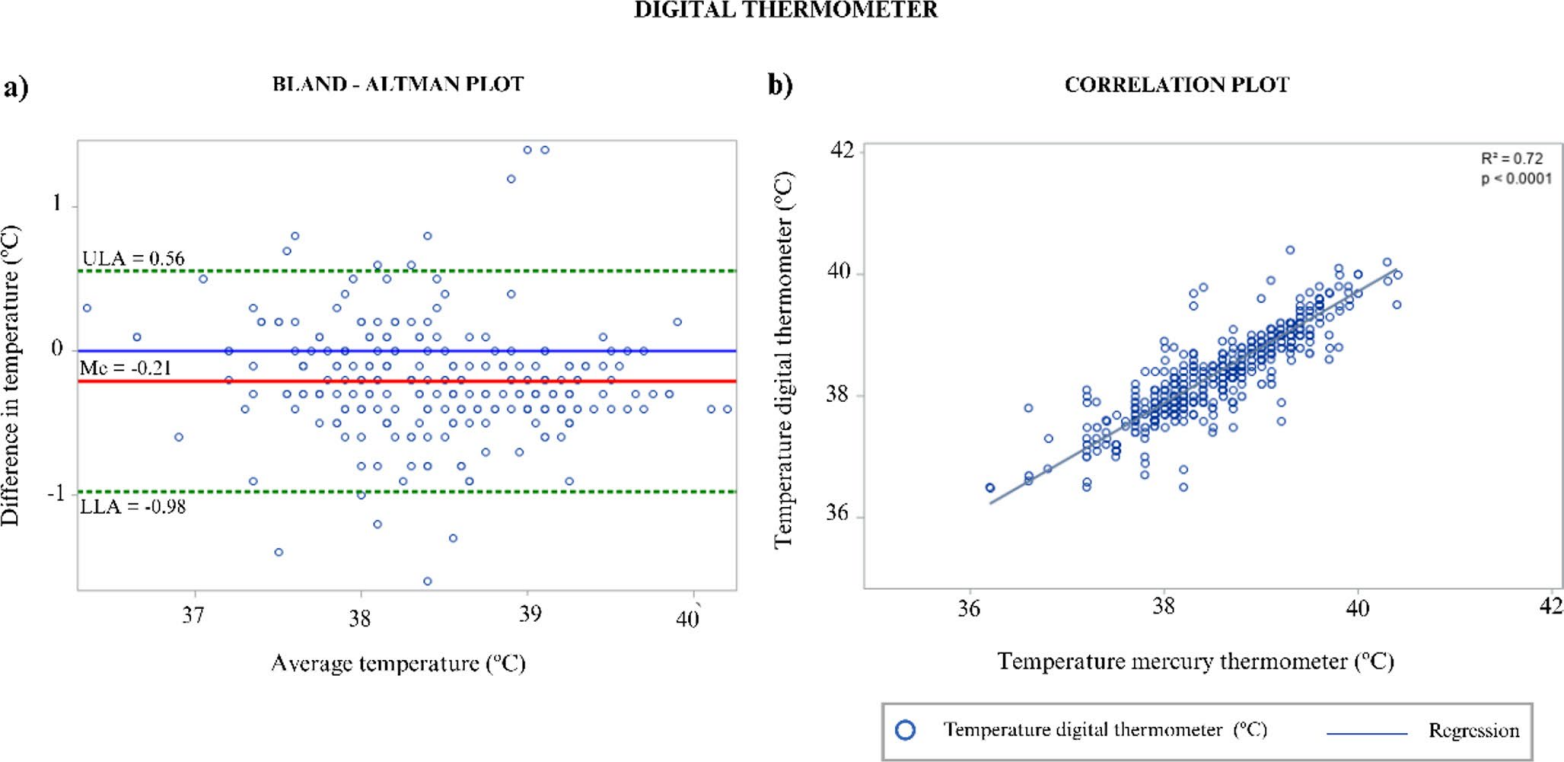
Digital thermometer graphics. (**a**) Bland-Altman plot of agreement between digital and mercury thermometers (∆ = MER – DIG). (**b**) Correlation plot with fitted linear regression line between DIG and MER (R² = 0.72, *p* < 0.0001)

Infrared thermometer readings were obtained at a fixed distance of 5 cm using a ruler (Fig. 3). Rectal measurements were performed after cutaneous measurements. A stable value was recorded when the same reading appeared in three consecutive scans; readings below 33.8 °C (“low”) were excluded.

All temperature measurements were performed by the same trained team across all animals and facilities. A single researcher was present at all evaluations and was exclusively responsible for acquiring all thermographic images. All operators received prior training to standardize animal handling, positioning, environmental conditions, and anatomical measurement sites.

### Statistical analysis

Statistical analyses were performed using SAS software (v9.4). Normality was assessed using the Shapiro–Wilk test. Pearson’s correlation was used for normally distributed data, and Spearman’s rank correlation was applied otherwise. Agreement was evaluated using Bland–Altman analysis, and proportional bias was assessed by linear regression. Clinical acceptability was defined as a standard deviation of differences < 0.5 °C and a mean difference within ± 0.3 °C, based on Burfeind et al. (2010). As the mercury thermometer was considered the reference method, agreement analyses were primarily focused on comparisons between each alternative device and the reference standard.

## Results

A total of 238 temperature readings per device were obtained. Two animals were excluded during the fifth afternoon session due to early discharge. The minor data loss observed (2 of 240 expected observations per device; <1%) was considered negligible and unlikely to affect the overall results.

The infrared thermometer did not measure skin temperatures below 33.8 °C, in which case the result “low” was obtained, which was considered missing. This data was obtained in all regions (FH 47%; RE 2.5%; LE 2.5%; RA 25.2%; LA 20.5%; PE 0.4%) more frequently in the morning (29%) than in the afternoon (4%). It was found that the maximum ambient temperature at which “low” was obtained was 24 °C.

Digital (DIG) and ecological (ECO) thermometers showed the closest agreement with mercury (MER), with similar mean and median temperatures. In contrast, infrared thermography (IRA) exhibited greater variability than infrared thermometers (INFRA) and rectal devices. Among IRA sites, the forehead (FHX) showed the widest interquartile range.

Pearson correlation coefficients and Bland–Altman agreement results are presented in Table 1. The strongest correlations with MER were observed for ECO (*r* = 0.90) and DIG (*r* = 0.85). Among INFRA measurements, the highest correlations were found at the right eye (RE; *r* = 0.61), left eye (LE; *r* = 0.63), and perineum (PE; *r* = 0.49). For IRA, moderate correlations were observed at the forehead (FHX; *r* = 0.65), right eye (REX; *r* = 0.64), and left eye (LEX; *r* = 0.66).

**Table 1:** Bland–Altman agreement statistics and correlation coefficients comparing infrared thermometer (INFRA) and infrared thermography (IRA) measurements with mercury rectal thermometers (MER) in cattle (*n* = 238)

		Region	Bland-Altman		Correlation	
			Me	SD	r	p-value
INFRA		FH	2.29	0.98	0.43	< 0.0001
		RE	1.01	0.67	0.61	< 0.0001
		LE	1.09	0.73	0.63	< 0.0001
		RA	1.74	0.95	0.28	< 0.0001
		LA	1.67	0.97	0.32	< 0.0001
		PE	0.67	0.72	0.49	< 0.0001
IRA		FHX	6.58	2.38	0.65	< 0.0001
		REX	1.79	0.71	0.64	< 0.0001
		LEX	1.83	0.74	0.66	< 0.0001
		RAX	4.13	1.41	0.44	< 0.0001
		LAX	4.13	1.54	0.41	< 0.0001
		PEX	1.72	1.12	0.49	< 0.0001

Me = mean difference between alternative method and mercury thermometer (°C); SD = standard deviation of the differences (°C); r = Pearson’s or Spearman’s correlation coefficient, selected according to data distribution; p-value = significance level associated with the correlation analysis; INFRA = infrared thermometer; IRA = infrared thermography; FH = forehead infrared thermometer; RE = right eye infrared thermometer; LE = left eye infrared thermometer; RA = right axilla infrared thermometer; LA = left axilla infrared thermometer; PE = perineum infrared thermometer; FHX = forehead infrared thermography; REX = right eye infrared thermography; LEX = left eye thermography; RAX = right axilla infrared thermography; LAX = left axilla infrared thermography; PEX = perineum infrared thermography

Despite these correlations, none of the INFRA or IRA measurements met the predefined agreement criteria for clinical replacement of MER. In contrast, ECO and DIG fulfilled all agreement thresholds, with low standard deviations and no systematic bias (ECO: SD = 0.33 °C, Me = 0.16 °C, *p* > 0.05; DIG: SD = 0.39 °C, Me = 0.21 °C, *p* > 0.05).

Bland–Altman analysis confirmed close agreement between ECO, DIG, and MER (Figs. 4 and 5), whereas INFRA and IRA consistently underestimated body temperature. For example, INFRA measurements at the forehead (FH) showed a mean difference of 2.29 °C (SD = 0.98 °C), while IRA measurements at the forehead (FHX) showed a mean difference of 6.58 °C (SD = 2.38 °C). Eye and perineum regions exhibited smaller mean differences than other sites, although values remained outside clinically acceptable limits.

Ambient temperature was positively correlated (*p* < 0.05) with all INFRA and IRA measurements, with the strongest associations observed at FHX (*r* = 0.84), FH (*r* = 0.78), LE (*r* = 0.78), and RE (*r* = 0.76). Relative humidity showed significant negative correlations but did not strongly influence any specific anatomical site.

## Discussion

There is limited evidence in veterinary medicine, particularly in ruminant species, evaluating mercury-free contact thermometers as substitutes for traditional mercury devices. In contrast, such alternatives have been extensively validated in human medicine, where digital and galinstan-based thermometers show clinically insignificant differences and acceptable agreement when compared with mercury thermometers (Davies et al. 1986; Smith 2003; Schreiber et al. 2013; Dante et al. 2021). Moreover, systematic reviews indicate that contact mercury-free thermometers provide more accurate estimates of core body temperature than peripheral infrared devices (Niven et al. 2015; Pecoraro et al. 2021).

Consistent with this body of evidence, the present study demonstrated that ecological (galinstan-based) and digital rectal thermometers are reliable alternatives for measuring body temperature in cattle. Both devices showed strong correlations with mercury thermometers (*r* ≥ 0.85), narrow limits of agreement, and no evidence of systematic bias, confirming their clinical equivalence to the traditional gold standard. Although veterinary data remain scarce, similar agreement between digital and mercury thermometers has been reported in animal studies (Rodrigues et al. 2021), reinforcing the suitability of these mercury-free devices for routine veterinary practice.

In contrast, neither infrared thermometers nor infrared thermography achieved clinically acceptable agreement with rectal temperature measured using mercury thermometers. Although moderate correlations were observed at ocular and perineal sites, the associated variability exceeded predefined clinical thresholds, indicating insufficient precision for replacing rectal measurements in cattle. These findings are consistent with previous veterinary and human studies reporting systematic underestimation of core body temperature by peripheral infrared methods (Cadioli et al. 2010; Salles et al. 2016; Peng et al. 2019; Pecoraro et al. 2021).

Among the cutaneous sites evaluated, the lacrimal region of the eyes showed the best overall performance for both infrared thermometry and thermography. Ocular temperature has been proposed as a potential non-invasive proxy for core temperature in several species (George et al. 2014; Gloster et al. 2011; Giannetto et al. 2022); however, inconsistent correlations reported in cattle and calves (Peng et al. 2019; Cantor et al. 2022) align with the present findings and highlight the limitations of ocular measurements as a standalone clinical tool.

Animal heterogeneity may have contributed to variability in infrared-based measurements. Differences in age, breed, hair coat characteristics, and management conditions influence skin thickness, vascularization, and thermoregulatory capacity (Hansen 2004; McManus et al. 2016). In the present study, animals were not clipped in order to replicate routine clinical conditions. Although such biological variability may increase dispersion in surface temperature values, all temperature measurements in this study were obtained from the same animals during the same evaluation sessions using multiple thermometric methods. This within-animal comparison minimizes the impact of individual biological variability and strengthens the robustness of the comparative assessment among temperature measurement techniques.

Infrared thermography generally yielded lower surface temperatures than infrared thermometers, likely reflecting differences in measurement distance and environmental exposure. Although measurements were conducted in shaded areas without direct solar radiation and with minimal airflow, infrared-based techniques remained sensitive to ambient conditions. This sensitivity was further evidenced by the significant correlations observed between environmental temperature and all infrared and thermographic measurements, confirming that surface temperature is more influenced by environmental variation than rectal temperature (Church et al. 2014; Peng et al. 2019). Environmental temperature and relative humidity were incorporated into thermographic image calibration; however, as the aim of this study was to evaluate device performance relative to the mercury thermometer under routine field conditions, environmental influence was considered part of the intrinsic performance of infrared-based methods, and no additional statistical adjustment was applied.

An additional limitation of infrared thermometry was the frequent occurrence of “low” readings (< 33.8 °C), particularly during morning measurements and in regions such as the forehead and axillae. This pattern may reflect lower physiological body temperature upon waking (Dukes 2006), combined with lower ambient temperatures during early hours (median 20 °C between 7:00–9:00 a.m. versus 24.6 °C between 1:00–3:00 p.m.) (Baura 2021). This limitation reduces the applicability of infrared thermometers under field conditions and further constrains their use for routine clinical assessment of body temperature in cattle.

Taken together, these findings emphasize the need for species-specific validation of non-invasive temperature assessment methods. While ecological and digital rectal thermometers are confirmed as clinically reliable mercury-free alternatives, infrared thermometry and thermography should not yet replace rectal measurements in cattle. Nevertheless, ocular and perineal regions remain promising targets for future investigations conducted under more controlled environmental conditions.

## Conclusion

Digital and ecological rectal thermometers were shown to be accurate and clinically reliable alternatives to mercury thermometers for measuring body temperature in cattle, with strong correlations and narrow limits of agreement supporting their safe adoption in veterinary practice.

In contrast, infrared thermometry and thermography did not achieve clinically acceptable agreement with mercury thermometers at any anatomical site. Although eye and perineum measurements showed relatively better performance, their accuracy remains insufficient for standalone clinical use.

Further studies under more controlled environmental conditions and with larger populations are required to refine the application of infrared techniques in cattle.

## Data Availability

The datasets generated and analyzed during the current study are available in the Mendeley Data repository: Pinheiro, Ana Carolina (2025), Bovine Thermometry, Mendeley Data, V1, 10.17632/vck3bxgmzp.
